# Gyogamdan, a Traditional Medicine Prescription, Ameliorated Dermal Inflammation and Hyperactive Behavior in an Atopic Dermatitis Mouse Model Exposed to Psychological Stress

**DOI:** 10.1155/2021/6687513

**Published:** 2021-03-30

**Authors:** Ly Thi Huong Nguyen, Uy Thai Nguyen, Min-Jin Choi, Tae-Woo Oh, In-Jun Yang, Heung-Mook Shin

**Affiliations:** ^1^Department of Physiology, College of Korean Medicine Dongguk University, Gyeongju 38066, Republic of Korea; ^2^Korean Medicine-Application Center, Korea Institute of Oriental Medicine, Daegu, Republic of Korea

## Abstract

Psychological stress (PS) plays a significant role as an aggravating factor in atopic dermatitis (AD). The traditional medicine prescription, Gyogamdan, has been used to treat chest discomfort and mood disorders caused by PS. This study investigated the effects of an ethanolic extract of Gyogamdan (GGDE) on stress-associated AD models and the underlying mechanisms. 2,4-Dinitrochlorobenzene- (DNCB-) treated BALB/c mice were exposed to social isolation (SI) stress. The effects of orally administered GGDE (100 or 500 mg/kg) were evaluated by ELISA, western blotting, and an open field test (OFT). SI stress exaggerated the skin inflammation and induced locomotor hyperactivity in the AD mouse model. GGDE reduced the levels of IgE, TNF-*α*, IL-13, eotaxin, and VEGF and mast cell/eosinophil infiltration and prevented the decreases in the levels of involucrin and loricrin in the skin. GGDE also suppressed the SI-induced increases in corticotropin-releasing hormone (CRH), adrenocorticotropic hormone (ACTH), and corticosterone (CORT) in socially isolated AD mice. Furthermore, GGDE reduced traveling distances and mean speed significantly in the OFT. The *in vitro* experiments were performed using HaCaT, HMC-1, PC12, and BV2 cells. In the TNF-*α*/IFN-*γ*- (TI-) stimulated HaCaT cells, GGDE decreased the thymus and activation-regulated chemokine (TARC) and macrophage-derived chemokine (MDC) production significantly by inhibiting p-STAT1 and NF-*κ*B signaling. GGDE also reduced VEGF production in HMC-1 cells stimulated with CRH/substance P (SP) by inhibiting p-ERK signaling pathway. GGDE increased the cell viability significantly and suppressed apoptosis in CORT-stimulated PC12 cells. Moreover, GGDE suppressed the LPS-induced production of NO, TNF-*α*, IL-1*β*, and IL-6 in BV2 cells. These results suggest that GGDE might be useful in patients with AD, which is exacerbated by PS.

## 1. Introduction

Individuals differ in terms of their perception and adaptation to psychological stress (PS). Some individuals are PS-sensitive and prone to develop PS-related pathologies, including mental disorders, whereas others are more PS-resilient [1]. A recent animal study reported that the responses to PS differ in normal and atopic dermatitis (AD) mice. PS did not affect normal mice but elicited significant changes in weight loss and anxiety-related behaviors in AD mice, suggesting that AD patients are likely to be vulnerable to PS [2].

AD and PS form a vicious cycle, each reinforcing the other negatively; AD symptoms can elicit PS, and PS can exacerbate AD symptoms [3, 4]. AD is an inflammatory skin disease characterized by redness, itching, swelling, and oozing bleeding. Sleep disturbances due to itching, anxiety about the possibility of scarring, and a chronic relapsing course can cause PS in AD patients [3, 5]. AD patients showed higher rates of stress-related disease, anxiety, and depression than healthy people [6]. Therefore, PS management in AD patients appears to be a promising area with considerable potential for treatment and management [7, 8].

The responses of AD patients to PS occur through coordination with the hypothalamus-pituitary-adrenal (HPA) axis. PS activates the HPA axis to stimulate systemic stress hormone production, including corticotropin-releasing hormone (CRH), adrenocorticotropic hormone (ACTH), and glucocorticoids (GC, cortisol in humans and corticosterone in rodent; hereafter referred to as CORT). These stress hormones contribute to mast cell activation, increased skin vascular permeability, and decreased epidermal structural protein production in the skin [3, 9]. PS induces CRH secretion from the hypothalamus, which activates skin mast cells to increase vasodilation and vascular permeability [4]. HPA activity is regulated by a GC negative feedback from the adrenal cortex. On the other hand, prolonged chronic elevation of CORT by PS can damage the skin barrier function [5, 10]. Moreover, an abnormal HPA axis function by PS causes structural and functional changes in the prefrontal cortex, hippocampus, and amygdala regions of the brain, resulting in increased stress vulnerability and attention-deficit/hyperactivity disorder (ADHD) of AD patients [11].

According to Donguibogam, the Korean traditional medicine book, Gyogamdan (GGD) is a traditional medicine used to treat chest discomfort and mood disorders caused by psychological stress. GGD consists of *Cyperus rotundus* L. and *Poria cocos* (*Schw*.) Wolf, both of which have been reported to be effective in stress-induced mental and physical diseases [12, 13]. *Cyperus rotundus* L. exhibited *in vivo* anti-inflammatory effects without affecting gastrointestinal tract [14]. *Cyperus rotundus* L. was indicated in the herbal formula KAHF, which significantly alleviated skin severity and serum levels of inflammatory cytokines in AD patients [15]. A previous study demonstrated that a methanolic extract of *Poria cocos (Schw.)* Wolf exerted inhibitory effects on 12-O-tetradecanoylphorbol-13-acetate- (TPA-) induced dermatitis in mice model [16]. On the other hand, it is unclear if GGD has therapeutic potential in AD exaggerated by PS. Therefore, the present study evaluated the efficacy and possible action mechanism of an ethanolic extract of GGD (GGDE) on the inflammatory response in an AD-like mouse model exposed to PS. In addition, behavioral changes were also assessed to determine if GGDE regulates the psychiatric comorbidities in a murine model of AD after exposure to PS.

## 2. Materials and Methods

### 2.1. Chemicals and Reagents

High glucose Dulbecco's modified Eagle's medium (DMEM) was obtained from Welgene Inc. (Gyeongsangbuk, Korea), and Iscove's modified Dulbecco's medium (IMDM) acquired from Merck Millipore (Darmstadt, Germany). Fetal bovine serum (FBS) and antibiotics were purchased from Invitrogen Inc. (Carlsbad, CA, USA). 2,4-Dinitrochlorobenzene (DNCB), corticotropin-releasing hormone (CRH), substance P (SP), 1-thioglycerol, and dexamethasone (DEX) were procured from Sigma-Aldrich (St. Louis, MO, USA). Human VEGF and mouse IFN-*γ*, IL-1*β*, IL-6, IL-13, TNF-*α*, VEGF, eotaxin, and IgE ELISA kits were supplied by Koma Biotech Inc. (Seoul, Korea). Human MDC, TARC ELISA kits were obtained from R&D Systems (Minneapolis, MN, USA). The mouse CORT ELISA kits were purchased from Arigo Biolaboratories (Hsinchu, Taiwan), and Mouse CRH and ACTH ELISA kits were procured from LifeSpan BioSciences (Seattle, WA, USA). Antibodies for phospho-p38, phospho-JNK, phospho-ERK, phospho-STAT1, phospho-I-*κ*B-*α*, p38, JNK, ERK, STAT1, I-*κ*B-*α*, and NF-*κ*B were acquired from Cell Signaling Technology (Danvers, MA, USA). Antibodies against loricrin, involucrin, and lamin B2 were supplied by Abcam (Cambridge, MA, USA). HRP-conjugated anti-*β*-actin was obtained from Sigma-Aldrich (St. Louis, MO, USA).

### 2.2. Plant Preparation and Analysis

GGD was purchased from Kyung Hee University Hospital (Seoul, Republic of Korea). GGD (*Poria cocos (Schw.)* Wolf to *Cyperus rotundus* L. weight ratio, 1 : 2) (30 g) was extracted with 300 ml of aqueous EtOH (30%) at 70°C for 3 h. The extract was then filtered through Whatman #2 filter paper (Whatman International, Maidstone, UK), evaporated on a rotary vacuum evaporator, and freeze-dried (FD8508S, Ilshin, Busan, Republic of Korea) (yield 7.83% w/w). The resulting dried GGD was dissolved in distilled dimethyl sulfoxide and sterilized by passing it through a 0.22 *μ*m syringe filter (Sartorius, Goettingen, Germany) before use. Nootkatone, *α*-cyperone, ergosterol, dehydrotrametenolic acid, pachymic acid, and tumulosic acid were purchased from ChemFaces Biochemical Co. Ltd. (Wuhan, China). Sample component analysis and quantification of the six standards were performed using an HPLC 1290 system (Agilent, Santa Clara, CA, USA) at the Korea Basic Science Institute (Seoul). For the analysis of nootkatone, *α*-cyperone, and ergosterol, the extracted samples (10 *μ*l) were separated on Extend C18 (2.0 × 150 mm, 5 *μ*m, Agilent) column at a flow rate of 0.4 ml/min using water (A) and acetonitrile (B) as the mobile phases. The gradient used was as follows: 50–80% (B) for 5 min, 80–100% (B) for 5 min, and equilibration for 5 min. The column was operated at 25°C. Nootkatone, *α*-cyperone, and ergosterol were detected at UV wavelengths of 240 nm, 254 nm, and 282 nm, respectively. For the analysis of dehydrotrametenolic acid, pachymic acid, and tumulosic acid, extracted samples (10 *μ*l) were separated on a Kinetex C18 (4.6 × 250 mm, 5 *μ*m, Phenomenex) column at a flow rate of 0.8 ml/min using 0.1% phosphoric acid (A) and acetonitrile (B) as the mobile phases. The gradient used was as follows: 10–100% B for 15 min and equilibration for 10 min. The column temperature was 35°C. Dehydrotrametenolic acid was detected at a UV wavelength of 210 nm; pachymic acid and tumulosic acid were detected at a UV wavelength of 243 nm. The amounts of these compounds in GGDE were determined using standard calibration curves.

### 2.3. Animal Experiments

**Figure 1:**
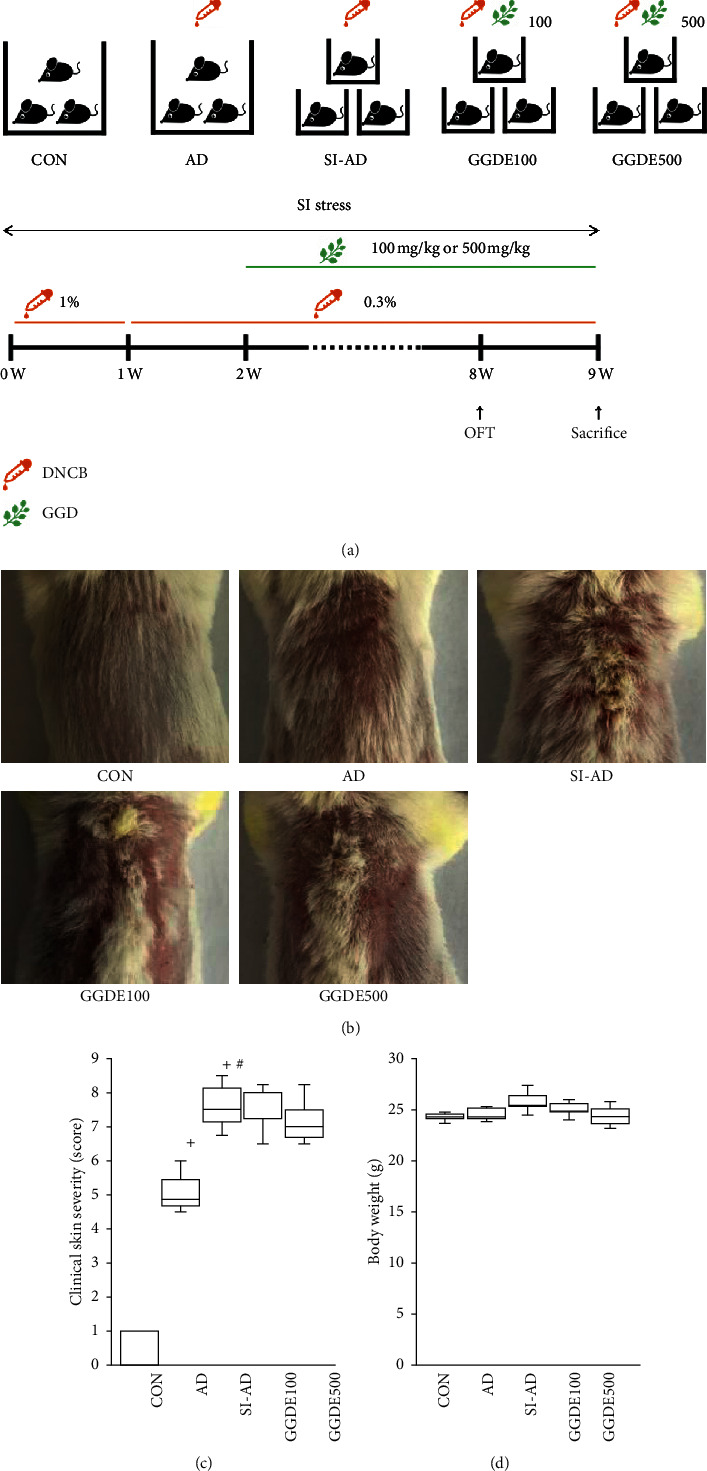
Effects of GGDE on AD mice exposed to PS. (a) Schematic diagram of the animal experiment. (b) Representative images of skin lesions. Clinical severity scores (c) and body weights (d) were measured. Results are presented as means ± SDs (*n* = 5 per experiment). +*P* < 0.05, vs. the CON group; ^#^*P* < 0.05, vs. the AD group; ^*∗*^*P* < 0.05, vs. the SI-AD group.

BALB/c (five-week-old male) mice were purchased from Koatech Lab Animal Inc. (Seoul). All animal experimental procedures were performed in accordance with the protocols approved by the Institutional Animal Care and Use Committee of Dongguk University (IACUC-2019-7). The mice were assigned randomly to five groups (*n* = 5 in each group): a treatment naïve control group (the CON group), DNCB only group (the AD group), DNCB plus social isolation group (the SI-AD group), DNCB plus PS plus 100 mg/kg/day of GGDE (the GGDE100 group), and DNCB plus PS plus 500 mg/kg/day of GGDE (the GGDE500 group). The animals were acclimated for one week before the experiments. All mice had free access to water and commercial pellet diet (5L79, PMI Nutrition, St Louis, MO, USA) Figure 1(a) presents a schematic diagram of the experimental schedule. Modified SI stress conditions were prepared according to a previous report of SI stress in a mouse model of allergic dermatitis [2]. The SI-AD group mice were isolated in acrylic cages (10 × 10 × 14 cm). The backs of the all mice were then shaved, and for sensitization, 200 *μ*l of 1% DNCB (dissolved in a 3 : 1 mixture of acetone and olive oil) was applied to all groups except CON group three times during the first week. Immediately after the treatment period, 200 *μ*l of 0.3% DNCB was applied to shaved dorsal areas three times per week for eight weeks in all groups except for the control group. From the 2nd week of the experiment, GGDE100 and GGDE500 group were administered 100 or 500 mg/kg of GGDE orally five times per week for seven weeks, respectively. The clinical skin severity score was measured for four symptoms: erythema/hemorrhage, edema, scarring/dryness, and erosion/excoriation, with the scoring system of 0 (no symptoms), 1 (mild), 2 (moderate), and 3 (severe). One day after the last treatment, the mice were sacrificed. The whole blood was collected, and serum was obtained by centrifugation (3000 × g, 15 min, 4⁰C).

### 2.4. Open Field Test (OFT)

The OFT was used to assess locomotor activities one week before sacrifice. Before testing, mice were acclimatized to a test room for two hours and then placed in a black box (measuring 40 × 40 × 40 cm), which was wiped down with 70% alcohol between tests to remove any odors. The total distances (cm) traveled, distances traveled in the peripheral zone and the central zone, and mean speeds (cm/s) were measured in a 10-minute test. Smart V3.0 software (Panlab Harvard Apparatus, MA, USA) was used to determine the total distance traveled, distance traveled in the central and peripheral zones, and the mean speed.

### 2.5. Histological and Immunohistochemical Examinations

After sacrifice, the dorsal skin samples were fixed immediately in 4% paraformaldehyde and embedded in paraffin. Sections were then cut and stained with hematoxylin and eosin (H&E), Congo red, or toluidine blue. The number of eosinophils and mast cells was counted in three randomly selected areas per sample at x200 using a Lionheart FX microscope equipped with Gen5 imaging software (BioTek Instruments, Winooski, VT, USA).

### 2.6. Cell Culture and Treatments

HaCaT cells (human keratinocyte cell line) were cultured in DMEM supplemented with 10% FBS, 1% penicillin-streptomycin, at 37°C in a 5% CO_2_ humidified environment. The medium was changed every two days during incubation, and the cells were made quiescent by starvation in serum-free medium for 24 h. Before stimulation with TI (TNF-*α* and IFN-*γ*, 10 ng/ml each) for the indicated time, the cells were pre-treated with GGDE (10, 100 *μ*g/ml) or DEX (10 *μ*M) for 1 h. The HMC-1 cells (a human mast cell line) were obtained from Merck Millipore (Darmstadt, Germany) and cultured in IMDM supplemented with 10% FBS, 1% penicillin-streptomycin, and 1.2 mM 1-thioglycerol at 37°C in a 5% CO_2_ humidified environment. HMC-1 cells were incubated with 10 *μ*M SP for 48 h and then with 1 nM CRH for 24 h. GGDE (10 or 100 *μ*g/ml) or DEX (10 *μ*M) was added 1 h before CRH. The PC12 cells (rat adrenal medullary pheochromocytoma cell line) were purchased from Korean Cell Line Bank (Seoul, Korea) and cultured in RPMI supplemented with 10% FBS, 1% penicillin-streptomycin, at 37°C in a 5% CO_2_ humidified environment. The PC12 cells were incubated with GGDE (10 or 100 *μ*g/ml) for 1 h and then stimulated with CORT (100 *μ*M) for 24 h. The BV2 cells (a mouse microglial cell line) were cultured in DMEM supplemented with 10% FBS and 1% penicillin-streptomycin at 37°C in a 5% CO_2_ humidified environment. BV2 microglial cells were pretreated with GGDE (10, 100, or 500 *μ*g/ml) or DEX (10 *μ*M) for 1 h and then stimulated with LPS (1 *μ*g/ml) for 24 h.

### 2.7. Cell Viability

The cell viability was determined using an XTT assay. After treating cells with GGDE (10, 100, 500, or 1000 *μ*g/ml) for 24 h, 50 *μ*l of the XTT solution was added and incubated for 4 h. The absorbances were measured at 450 nm (reference wavelength 650 nm) using a microplate reader (Molecular Devices, CA, USA).

### 2.8. Enzyme-Linked Immunosorbent Assay (ELISA)

Skin tissues were homogenized with tissue extraction reagent (Thermo Fisher Scientific, Vienna, Austria) and centrifuged at 10, 000 × g for 20 min. The supernatants were collected and made up to a protein concentration of 0.5 *μ*g/*μ*l. The protein levels in the skin tissues and cell culture supernatants and the IgE levels in the serum were measured using commercial ELISA kits. The absorbance was measured at 450–540 nm using an automated microplate reader (Molecular Devices CA, USA).

### 2.9. Western Blot Analysis

Skin tissues were homogenized using a tissue extraction reagent, centrifuged at 10, 000 × g for 20 min, and the supernatants were collected. HaCaT cells were washed with 1X PBS and then lysed with RIPA lysis buffer containing the protease and phosphatase inhibitors (Atto, Tokyo). After sonication, the cell lysates were centrifuged at 8000  × g for 15 min, and the supernatants were collected. The total protein levels were determined using the Bradford protein assay reagent (BioRad, CA, USA). Subsequently, 25–50 *μ*g of the total proteins were separated by 7.5–10% SDS-PAGE electrophoresis and transferred to PVDF membranes (Merck Millipore, Darmstadt, Germany). The proteins were then blocked with 5% skim milk in 1X PBS for 2 h at room temperature. They were then incubated with the primary antibodies, followed by the secondary antibody horseradish peroxidase-conjugated anti-IgG. The blots were detected by enhanced chemiluminescence (BioRad), and the band intensities of the proteins were quantified using GelPro V3.1 software (Media Cybernetics, MD, USA).

### 2.10. Cell Apoptosis Analysis

The percentage of apoptotic cells was determined using the Muse Annexin V and Dead Cell kit (Millipore, Billerica, MA, USA) according to the manufacturer's protocol. PC12 cells were incubated with GGDE (10, 100 *μ*g/ml) for 1 h and stimulated with CORT (100 *μ*M) for 24 h. Subsequently, 100 *μ*l of Annexin V and Dead Cell reagent and 100 *μ*l of a single cell suspension were mixed in a microtube and incubated in the dark for 20 min at room temperature. The cells were analyzed using a Muse Cell Analyzer (Millipore, Billerica, MA, USA).

### 2.11. Nitric Oxide (NO) Assay

The production of NO was measured using a Griess reagent assay. BV2 microglial cells were pre-treated with GGDE (10, 100, 500 *μ*g/ml) or DEX (10 *μ*M) for 1 h and stimulated with lipopolysaccharides (LPS, 1 *μ*g/ml) for 24 h. Subsequently, 100 *μ*l of supernatant was mixed with 100 *μ*l of Griess reagent at room temperature. The absorbances were measured at 540 nm using an automated microplate reader (Molecular Devices CA, USA).

### 2.12. Statistical Analysis

The data were analyzed using a Student's unpaired *t*-test. *P* values < 0.05 were considered significant. The *in vivo* results are presented as box and whisker plots with the medians as the middle lines, the 25^th^/75^th^ quartiles as the box edges, and the minimum/maximum as whiskers. The *in vitro* data are presented as the mean ± SDs of three independent experiments.

## 3. Results

### 3.1. Effects of GGDE on AD Mice Exposed to PS

The DNCB treatment caused AD-like symptoms, such as erythema and edema, which were exacerbated by SI stress. GGDE suppressed the SI-induced increases in skin severity score, but no significant difference was observed between the SI-AD and GGDE groups (Figures 1(b) and 1(c)). No mortality was noted in the GGDE groups. Treatment with GGDE at 100 and 500 mg/kg did not show significant changes in body weights compared to the CON group (Figure 1(d)).

### 3.2. Effects of GGDE on Dorsal Skin Lesions in AD Mice Exposed to PS

**Figure 2:**
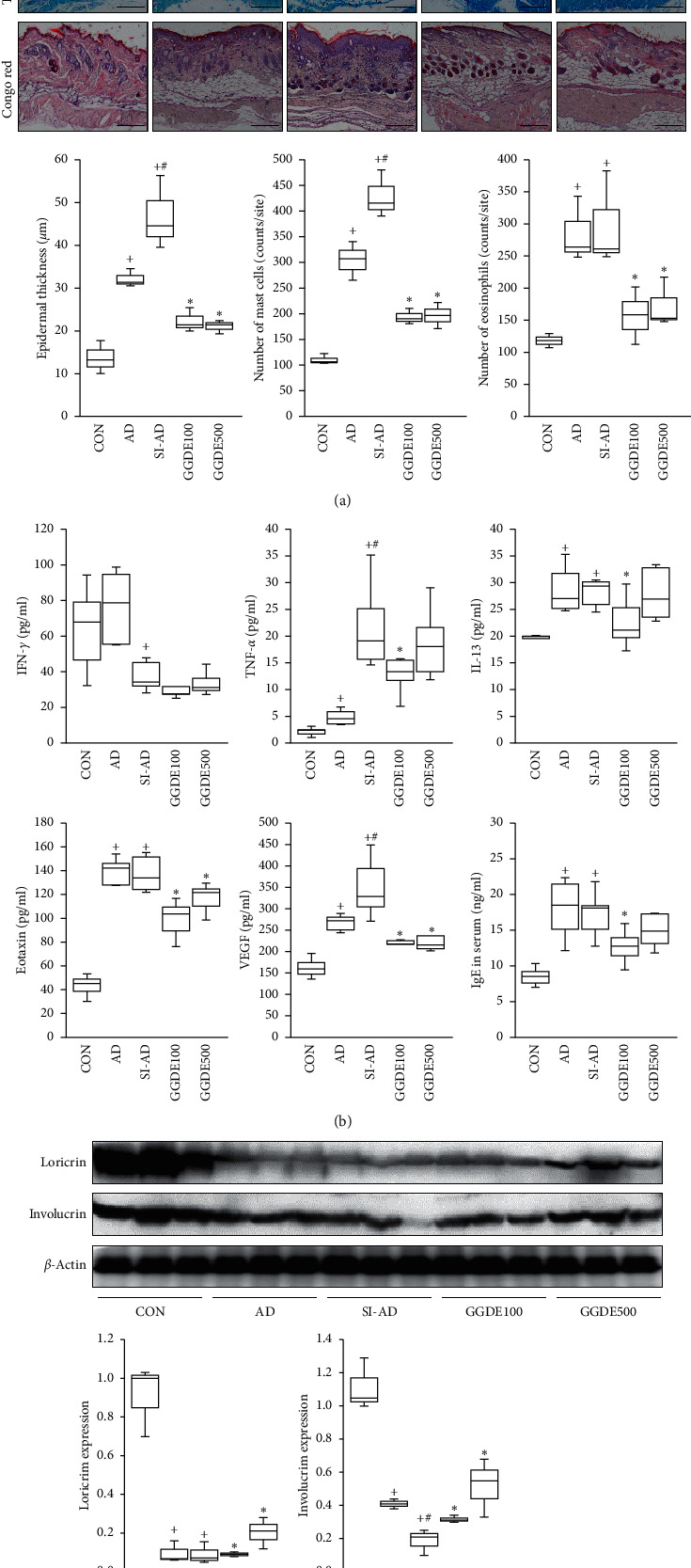
Effects of GGDE on the inflammatory response in AD mice exposed to PS. (a) The results of histological staining in the dorsal skin. Scale bar: 250 *μ*m. (b) Skin tissue levels of inflammatory mediators and serum IgE levels were measured. The results are presented as means ± SDs (*n* = 5 per experiment). (c) Effects of GGDE on the skin barrier protein expression in AD mice exposed to PS. Western blot of loricrin and involucrin in dorsal skin lesions. Results are presented as the means ± SDs (*n* = 3 per experiment). +*P* < 0.05, vs. the CON group; #*P* < 0.05, vs. the AD group; ^*∗*^*P* < 0.05, vs. the SI-AD group.

Marked eosinophil infiltrations were observed in the SI-AD mice, and the mast cell infiltrations and epidermal thickness were significantly higher in the SI-AD mice than the AD mice. On the other hand, epidermal thicknesses in the GGDE (100 and 500 mg/kg) groups were significantly smaller than in the SI-AD group. Eosinophil and mast cell infiltrations in lesions were less severe in the GGDE mice than in the SI-AD mice (Figure 2(a)). The TNF-*α*, IL-13, eotaxin, and VEGF levels in the skin lysates were all significantly higher in the SI-AD mice than in the CON mice. Interestingly, although the SI-AD mice had significantly higher levels of TNF-*α* and VEGF than the AD mice, they also had significantly lower levels of IFN-*γ*. In contrast, the levels of eotaxin and VEGF were lower in the GGDE-treated mice (100 and 500 mg/kg) than in the SI-AD mice, and the TNF-*α* and IL-13 levels were lower in the GGDE100 group than in the SI-AD group. Furthermore, mice in the AD and SI-AD groups had significantly higher serum IgE levels than the mice in the CON group, but the mice in the GGDE100 group had significantly lower IgE levels than those in the SI-AD group (Figure 2(b)). The negative effects of PS on the skin barrier function are well established [5, 10]. Loricrin and involucrin expression, which are positively associated with the skin barrier function, were significantly lower in the AD and SI-AD mice than in the CON mice. GGDE (100 and 500 mg/kg) prevented these decreases (Figure 2(c)).

### 3.3. Effects of GGDE on the HPA Axis and the Behavioral Changes of AD Mice Exposed to PS

**Figure 3:**
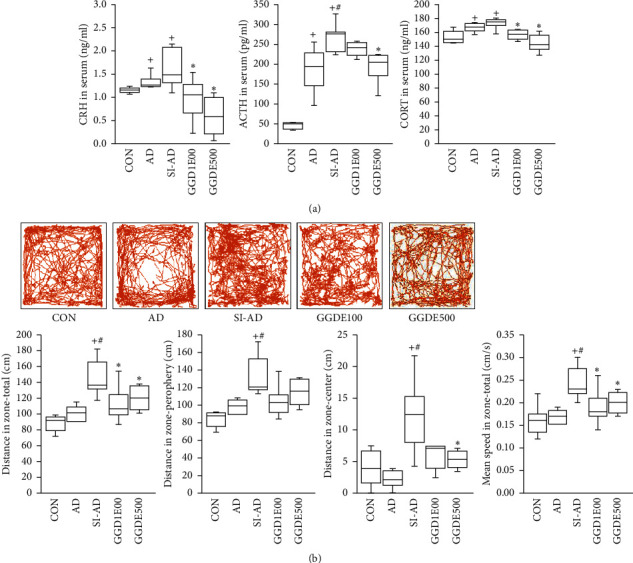
Effects of GGDE on hypothalamic-pituitary-adrenal (HPA) axis and behavioral changes in AD mice exposed to PS. (a) CRH, ACTH, and CORT levels in serum. (b) Behavior in the open field test. Representative open field test track sheets. Total distances (cm) traveled, distances traveled in the peripheral zone and central zone, and mean speeds (cm/s) in the 10-minute test. The results are presented as the means ± SDs (*n* = 5 per experiment). +*P* < 0.05, vs. the CON group; #*P* < 0.05, vs. the AD group; ^*∗*^*P* < 0.05, vs. the SI-AD group.

The serum CRH, ACTH, and CORT levels were measured to assess the HPA axis reactivity to PS. The AD and SI-AD mice had significantly higher serum CRH, ACTH, and CORT levels than the CON mice, and the SI-AD mice had significantly higher levels of ACTH than the AD mice. On the other hand, the serum CRH, ACTH, and CORT levels were reduced significantly by GGDE500, whereas GGDE100 reduced only the CRH and CORT levels (Figure 3(a)). PS can also be assessed by the behavioral indicators [17]. OFT was performed to assess the effect of GGDE on the behaviors of AD mice exposed to PS. The distances traveled in the central, peripheral, and total zones, and mean speeds in OFT were significantly greater for the SI-AD mice than the CON and AD mice. On the other hand, the central and total zone distances and mean speeds were decreased significantly in the GGDE500 mice. The distances traveled in the total zones and mean speeds were significantly lower in the GGDE100 mice (Figure 3(b)).

### 3.4. Effects of GGDE on Inflammatory Response in HaCaT and HMC-1 Cells

**Figure 4:**
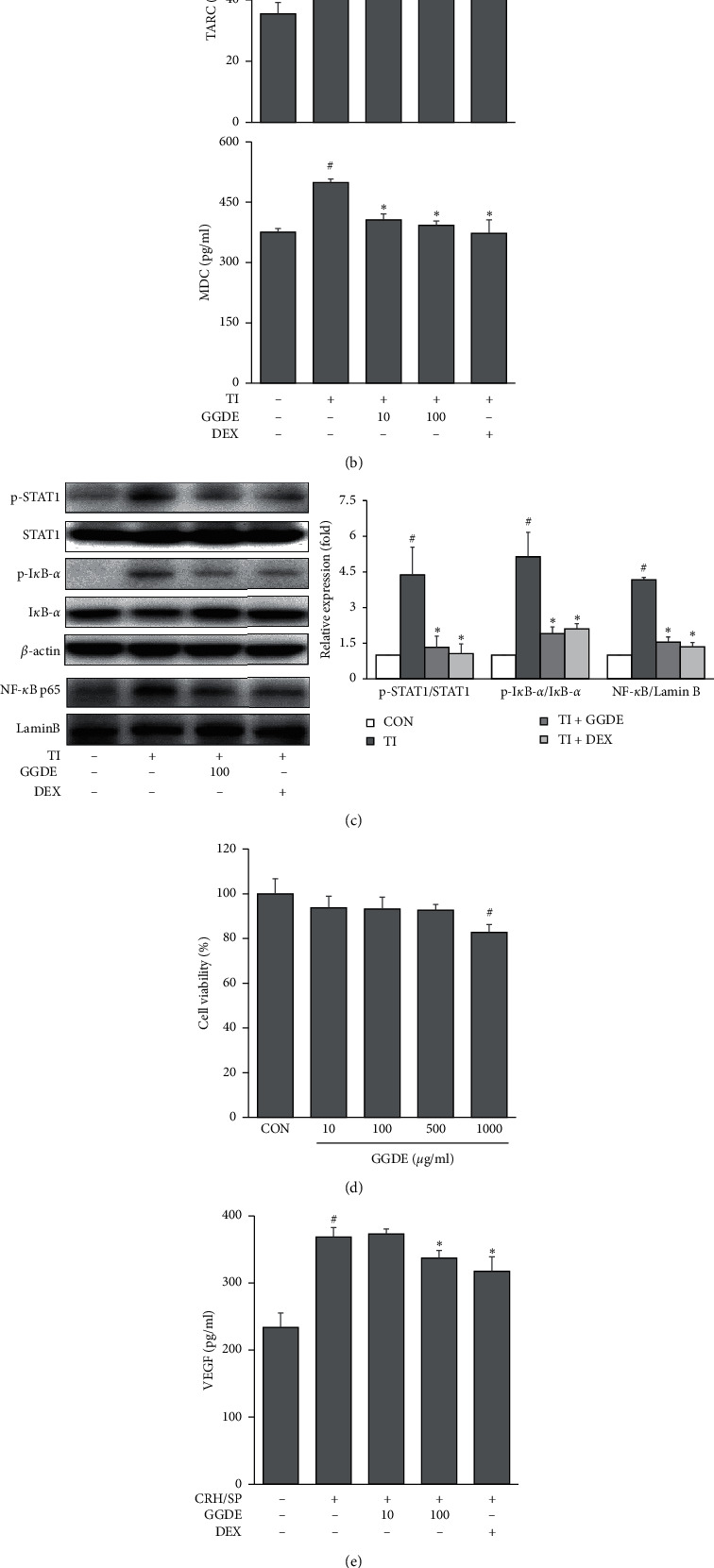
Effects of GGDE on inflammatory mediator release in HaCaT and HMC-1 cells. (a) The HaCaT cell viabilities were assessed using XTT assays. (b) Effects of GGDE on TI (TNF-*α* and IFN-*γ*)-induced TARC and MDC productions. (c) Western blot of p-STAT1, STAT1, p-I*κ*B-*α*, I*κ*B-*α*, *β*-actin, NF-*κ*B p65, and lamin B in HaCaT cells. (d) HMC-1 cell viabilities were assessed using XTT assays. (e) Effects of GGDE on CRH/SP-induced VEGF production. (f) Western blot of p-ERK, ERK, p-p38, p38, p-JNK, JNK, and *β*-actin in HMC-1 cells. Dexamethasone (DEX) was used as a positive control. The results are presented as means ± SDs (*n* = 3 per experiment). #*P* < 0.05, vs. CON; ^*∗*^*P* < 0.05, vs. T/I-treated cells; *∗P* < 0.05, vs. CRH/SP-treated cells.

The *in vitro* effects of GGDE were examined to understand its action mechanism. Initially, the cytotoxic effects were determined using XTT assays, and subsequent experiments were carried out at nontoxic concentrations (Figures 4(a) and 4(d)). TI (TNF-*α* and IFN-*γ*, 10 ng/ml each) stimulation increased the Th2 chemokine (TARC, MDC) levels in the HaCaT supernatants, but these increases were suppressed by GGDE (10 and 100 *μ*g/ml). The STAT1 and NF-*κ*B pathways play important roles as signaling mediators of the cellular responses to extracellular signals associated with the inflammatory response in AD [18]. GGDE (100 *μ*g/ml) significantly inhibited the TI upregulated p-STAT1 expression and NF-*κ*B p65 nuclear translocation by downregulating I*κ*B-*α* phosphorylation (Figures 4(b) and 4(c)). The HMC-1 cells were treated with CRH and SP (CRH/SP) to mimic the PS-exaggerated AD condition [4]. GGDE (100 *μ*g/ml) decreased CRH/SP-induced VEGF production and ERK phosphorylation in the HMC-1 cells, indicating that the therapeutic effect of GGDE involves mast cell suppression (Figure 4(e) and 4(f)).

### 3.5. Effects of GGDE on CORT-Induced Neurotoxicity in PC12 Cells

**Figure 5:**
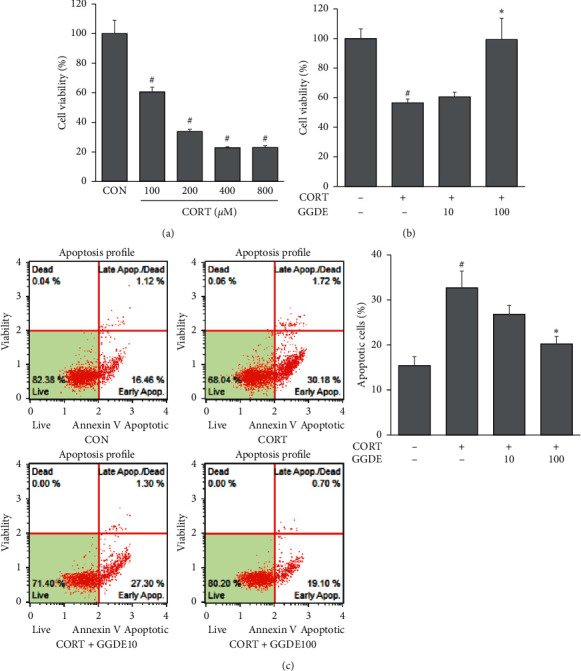
Effects of GGDE on CORT-induced neurotoxicity in PC12 cells. (a) Effects of CORT on the viability of PC12 cells. (b) Effects of GGDE on cell viability in CORT-stimulated PC12 cells. (c) Effects of GGDE on apoptosis rate in CORT-stimulated PC12 cells. The percentage of apoptotic cells was measured using Muse Annexin V and Dead Cell kit. Results are presented as means ± SDs (*n* = 3 per experiment). #*P* < 0.05, vs. CON; ^*∗*^*P* < 0.05, vs. CORT-treated cells.

As shown in Figure 5(a), CORT reduced the viability of PC12 cells significantly. When treated with 100 *μ*M CORT for 24 h, the cell viability decreased to approximately 60%; therefore, this concentration was used in subsequent experiments. Pretreatment with GGDE (100 *μ*g/ml) increased cell viability in CORT-treated PC12 cells significantly (Figure 5(b)). Moreover, CORT induced apoptosis in PC12 cells, which was suppressed by the GGDE pretreatment (Figure 5(c)).

### 3.6. Effects of GGDE on LPS-Induced Neuroinflammation in BV2 Cells

**Figure 6:**
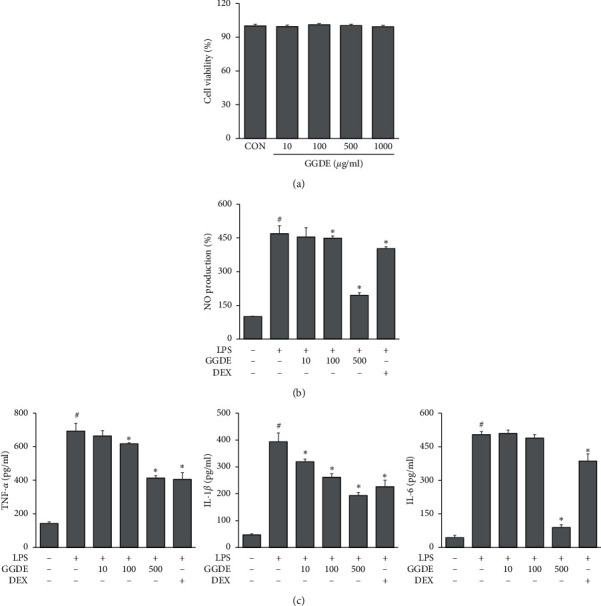
Effects of GGDE on LPS-induced neuroinflammation in BV2 cells. (a) Effects of GGDE on the viability of BV2 cells. (b) Effects of GGDE on NO production in LPS-stimulated BV2 cells. (c) Effects of GGDE on the production of TNF-*α*, IL-1*β*, and IL-6 in LPS-stimulated BV2 cells. Results are presented as means ± SDs (*n* = 3 per experiment). #*P* < 0.05, vs. CON; ^*∗*^*P* < 0.05, vs. LPS-treated cells.

GGDE did not show any cytotoxic effects on BV2 cells (Figure 6(a)); therefore, GGDE at concentrations of 10, 100, and 500 *μ*g/ml was used for subsequent experiments. NO secreted by activated microglia plays an important role in neuroinflammation and neuronal death [19]. As shown in Figure 6(b), LPS increased NO production in BV2 cells, but this increase was reduced significantly by pretreatment with GGDE (100, 500 *μ*g/ml). The levels of inflammatory cytokines TNF-*α*, IL-1*β*, and IL-6, were upregulated by LPS stimulation (Figure 6(c)). GGDE (100 or 500 *μ*g/ml) suppressed TNF-*α* and IL-1*β* production, but the IL-6 level was decreased only by a pretreatment with GGDE at 500 *μ*g/ml (Figure 6(c)).

### 3.7. Quantitative Determination of the Constituents in GGDE

**Figure 7:**
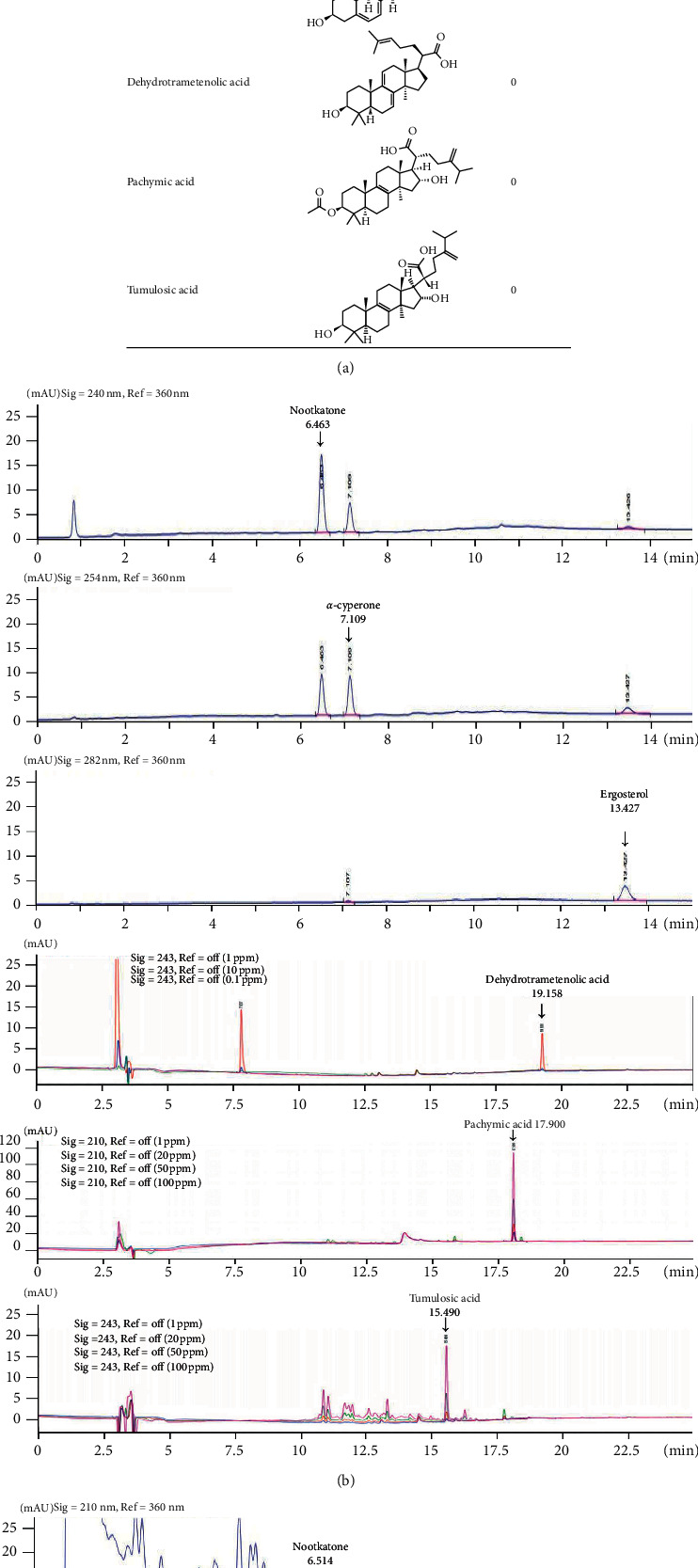
(a) Composition and chemical components in GGDE. (b) HPLC chromatograms of commercial standards for detecting nootkatone and *α*-cyperone, ergosterol, dehydrotrametenolic acid, pachymic acid, and tumulosic acid. (c) HPLC chromatograms of GGDE for the detection of nootkatone and *α*-cyperone.

The active constituents of GGD are nootkatone, *α*-cyperone, ergosterol, dehydrotrametenolic acid, pachymic acid, and tumulosic acid (Figure 7(a)) [20, 21]. Therefore, the amounts of these compounds in GGDE were determined by HPLC. Figures 7(b) and 7(c) show representative chromatogram patterns of the standard compounds and GGDE. The retention times of nootkatone and *α*-cyperone were 6.514 and 7.126 min, respectively. The concentrations of these two components in GGDE were 0.008 and 0.422 *μ*g/mg, respectively (Figure 7(a)). Ergosterol, dehydrotrametenolic acid, pachymic acid, and tumulosic acid were not detected in GGDE.

## 4. Discussion

SI stress is a well-characterized type of PS used to investigate the impact of PS on AD [7]. In the present study, SI stress increased DNCB-induced mast cell infiltration, TNF-*α*, and VEGF production in dorsal skin lesions. SI stress reduced the production of IFN-*γ* in SI-AD mice to levels significantly lower than those of CON mice. These findings concur with studies that found that PS induced Th1/Th2 imbalance [3, 9]. Moreover, the OFT results showed that SI could be a potential factor in accelerating ADHD symptoms, such as hyper-locomotor activity in AD.

In AD, keratinocytes activated by allergens and proinflammatory cytokine secrete chemokines that induce immune cell infiltration of the skin lesions. The immune cell infiltration increases the levels of proinflammatory cytokines and maintains a chronic inflammation [22, 23]. GGDE decreased TNF-*α*, IL-13, eotaxin, IgE production, and reduced eosinophils infiltration in SI-AD mice. In addition, treatment of human keratinocyte HaCaT cells with GGDE suppressed Th2-attracting chemokines TARC and MDC production and decreased STAT-1 and NF-*κ*B activation. In particular, GGDE500 *μ*g/ml and DEX, which was used as a positive control in this study, had a similar inhibitory effect on MDC production. These results suggest that GGDE can suppress the proinflammatory mediator and infiltration of immune cells by blocking the chemokine production in AD exposed to PS.

The PS-elicited secretion of CRH can enhance the production of VEGF in mast cells; hence, it can trigger or exacerbate AD [24]. The extent of mast cell infiltration and VEGF levels in SI-AD mice were significantly higher than in AD mice. Oral administration of GGDE reduced the serum CRH levels in a dose-dependent manner. Together with the present results, it was hypothesized that GGDE might inhibit VEGF production in mast cells increased by CRH stimulation. The effects of GGDE on CRH/SP-treated HMC-1 cells were evaluated to understand the actions of GGDE at the cellular level better. SP can increase the expression of CRH receptor-1 in mast cells, and the application of SP and CRF to HMC-1 can mimic PS-associated AD conditions [4]. Consistent with the *in vivo* data, GGDE inhibited VEGF production in CRH/SP-treated HMC-1 cells by inhibiting p-ERK. These results suggested that the therapeutic effects of GGDE do not depend on the direct regulations of inflammatory mediators but on the indirect regulation of inflammatory response by regulating the release of CRH.

The rising levels of CORT have negative effects on skin barrier function and stratum corneum integrity [3, 25]. Increased CORT levels and decreased expression of the epidermal differentiation-related proteins were found in SI-AD mice, which were prevented by GGDE. A previous study showed that a natural ingredient, hesperidin, prevented GC-impaired skin barrier homeostasis by increasing the epidermal differentiation-related proteins [26]. These results suggest that GGDE can prevent skin barrier disruption by inflammatory cytokines and by CORT.

Patients with AD had a higher incidence of ADHD than non-AD patients [27, 28]. The underlying mechanism of this comorbidity is that AD-related PS causes hyperactivity in the HPA axis, leading to neurotoxicity of the hippocampus or prefrontal cortex. [11, 29]. High concentrations of CORT impair the HPA negative feedback loop, and prolonged high CORT levels induced further neuronal damage [5, 30]. Previous studies showed that hyperactivity is accompanied by increases in the circulating ACTH levels [31, 32]. Neuroinflammation, particularly microglial activation, has also been suggested to mediate stress-induced psychiatric disorders [33]. These results have shown that the oral administration of GGDE decreased the CORT and ACTH levels as well as the hyper-locomotion activity in SI-AD mice. In addition, GGDE has a protective effect on CORT-induced neurotoxicity in PC12 cells, which may be related to the inhibition of apoptosis. GGDE inhibited LPS-induced microglial activation by suppressing the production of NO, TNF-*α*, IL-1*β*, and IL-6 in BV2 cells. These findings show that GGDE may be a new treatment option for treating ADHD, the psychiatric comorbidity of stress-susceptible AD patients.

GGD is a traditional herbal prescription comprised of *Cyperus rotundus* L. and *Poria cocos (Schw.)* Wolf., which showed antiallergic and anti-inflammatory effects in previous studies [34, 35]. Topical application of *C. rotundus* extract suppressed infiltration of inflammatory cells and ear edema in TPA-induced skin inflammation in mice [36]. A preclinical study demonstrated that oral administration of *P. cocos* extract ameliorated DNCB-induced AD-like symptoms in mice by modulating the production of Th2 cytokines and the generation of Treg cells [37]. In the present study, the combination of *C. rotundus* and *P. cocos* in GGDE not only did have inhibitory effects on skin inflammation but also improved psychiatric comorbidity in AD mice exposed to SI stress, suggesting synergistic effects of this combination for the treatment of AD. HPLC showed that GGDE contains two major chemical constituents: *α*-cyperone and nootkatone. A herbal formula containing *α*-cyperone was reported to have potent antidepressant effects by regulating prefrontal cortex activation in a mouse model of depression induced by PS [38]. In contrast, nootkatone was reported to inhibit the Th2-attracting chemokines TARC and MDC in keratinocytes [39]. Overall, *α*-cyperone and nootkatone might at least be partially responsible for regulating the inflammation, HPA axis alteration, and hyperactive behavior in PS-exacerbated AD.

The present study had some limitations. Because there was no adequate positive control available for the AD-like animal model worsened by PS, DEX was used as a positive control to compare the anti-inflammatory efficacy only in *in vitro* experiments. The current study was conducted to assess the effects of an aqueous ethanolic extract of GGD in an animal model. Therefore, further experiments will be needed to find appropriate concentrations to manage clinical symptoms and signs more effectively. Despite the limitations of the current study, these results provide a clue for treating patients with AD exacerbated by PS.

## 5. Conclusion

GGDE ameliorates the cutaneous inflammation, skin barrier disruption, HPA axis activation, and hyper-locomotor activity in AD mice exposed to SI. GGDE could inhibit chemokines production in keratinocytes and VEGF release in mast cells treated with SP and CRH. Moreover, GGDE has protective effects against CORT-induced neurotoxicity in PC12 cells and LPS-induced neuroinflammation in BV2 cells. These findings suggest that GGDE is a potential candidate as a treatment for patients with AD exacerbated by PS.

## Data Availability

The data used to support the findings of this study are included within the article.

## Abbreviations

HPA: Hypothalamic-pituitary-adrenal
IL: Interleukin
MDC: Macrophage-derived chemokine
TARC: Thymus- and activation-regulated chemokine
VEGF: Vascular endothelial growth factor.

## References

[1] Jakovcevski M., Schachner M., Morellini F. (2008). Individual variability in the stress response of C57BL/6J male mice correlates with trait anxiety. *Genes, Brain and Behavior*.

[2] Kitagaki H., Hiyama H., Kitazawa T., Shiohara T. (2014). Psychological stress with long-standing allergic dermatitis causes psychodermatological conditions in mice. *Journal of Investigative Dermatology*.

[3] Lin T.-K., Zhong L., Santiago J. (2017). Association between stress and the HPA Axis in the atopic dermatitis. *International Journal of Molecular Sciences*.

[4] Sur B., Lee B., Yoon Y. S. (2017). Extract of polygala tenuifolia alleviates stress-exacerbated atopy-like skin dermatitis through the modulation of protein kinase A and p38 mitogen-activated protein kinase signaling pathway. *International Journal of Molecular Sciences*.

[5] Park G., Lee S. H., Oh D. S., Kim Y. U. (2017). Melatonin inhibits neuronal dysfunction-associated with neuroinflammation by atopic psychological stress in NC/Nga atopic-like mouse models. *Journal of Pineal Research*.

[6] Silverberg J. I. (2019). Comorbidities and the impact of atopic dermatitis. *Annals of Allergy, Asthma & Immunology*.

[7] Jiang J., Yamaguchi T., Funakushi N. (2009). Oral administration of yokukansan inhibits the development of atopic dermatitis-like lesions in isolated NC/Nga mice. *Journal of Dermatological Science*.

[8] Li Y., Chen L., Du Y., Huang D., Han H., Dong Z. (2016). Fluoxetine ameliorates atopic dermatitis-like skin lesions in BALB/c mice through reducing psychological stress and inflammatory response. *Frontiers in Pharmacology*.

[9] Arck P. C., Slominski A., Theoharides T. C., Peters E. M. J., Paus R. (2006). Neuroimmunology of stress: skin takes center stage. *Journal of Investigative Dermatology*.

[10] Choe S. J., Kim D., Kim E. J. (2018). Psychological stress deteriorates skin barrier function by activating 11beta-hydroxysteroid dehydrogenase 1 and the HPA axis. *Scientific Reports*.

[11] Buske-Kirschbaum A., Schmitt J., Plessow F., Romanos M., Weidinger S., Roessner V. (2013). Psychoendocrine and psychoneuroimmunological mechanisms in the comorbidity of atopic eczema and attention deficit/hyperactivity disorder. *Psychoneuroendocrinology*.

[12] Choi J. E., Park D.-M., Chun E. (2017). Control of stress-induced depressive disorders by So-ochim-tang-gamibang, a Korean herbal medicine. *Journal of Ethnopharmacology*.

[13] Sun H. Y., Li Q., Liu Y. Y. (2017). Xiao-yao-san, a Chinese medicine formula, ameliorates chronic unpredictable mild stress induced polycystic ovary in rat. *Frontiers in Physiology*.

[14] Ahmad M., MahayRookh R., Rehman A. B (2014). Assessment of anti-inflammatory, anti-ulcer and neuro-pharmacological activities of Cyperus rotundus Linn. *Pakistan Journal of Pharmaceutical Sciences*.

[15] Koh D. J., Kim Y. H., Kim D. G., Lee J. Y., Lee K. T. (2008). Evaluation of the atopic dermatitis-mitigating and anti-inflammatory effects of Kyung hee allergic disease herbal formula (KAHF). *Food Science and Biotechnology*.

[16] Kaminaga T., Yasukawa K., Takido M., Tai T., Nunoura Y. (1996). Inhibitory effect of Poria cocos on 12-O-tetradecanoylphorbol-13-acetate-induced ear oedema and tumour promotion in mouse skin. *Phytotherapy Research*.

[17] Beery A. K., Kaufer D. (2015). Stress, social behavior, and resilience: insights from rodents. *Neurobiology of Stress*.

[18] Kwon D.-J., Bae Y.-S., Ju S. M. (2012). Casuarinin suppresses TARC/CCL17 and MDC/CCL22 production via blockade of NF-*κ*B and STAT1 activation in HaCaT Cells. *Biochemical and Biophysical Research Communications*.

[19] Yuste J. E., Tarragon E., Campuzano C. M., Ros-Bernal F. (2015). Implications of glial nitric oxide in neurodegenerative diseases. *Frontiers in Cellular Neuroscience*.

[20] Ríos J. L. (2011). Chemical constituents and pharmacological properties of Poria cocos. *Planta Medica*.

[21] Nuryana F. I., Chozin M., Guntoro D. (2019). Hight performance liquid chromatography analysis for *α* cyperone and nootkatone from the tuber of nutsedge (Cyperus rotundus L.) in the tropics. *Rasayan Journal of Chemistry*.

[22] Jovanovic K., Siebeck M., Gropp R. (2014). The route to pathologies in chronic inflammatory diseases characterized by T helper type 2 immune cells. *Clinical & Experimental Immunology*.

[23] Brandt E. B., Sivaprasad U. (2011). Th2 cytokines and atopic dermatitis. *Journal of Clinical and Cellular Immunology*.

[24] Asadi S., Alysandratos K.-D., Angelidou A. (2012). Substance P (SP) induces expression of functional corticotropin-releasing hormone receptor-1 (CRHR-1) in human mast cells. *Journal of Investigative Dermatology*.

[25] Maarouf M., Maarouf C. L., Yosipovitch G., Shi V. Y. (2019). The impact of stress on epidermal barrier function: an evidence‐based review. *British Journal of Dermatology*.

[26] Man G., Mauro T. M., Kim P. L. (2014). Topical hesperidin prevents glucocorticoid-induced abnormalities in epidermal barrier function in murine skin. *Experimental Dermatology*.

[27] Yaghmaie P., Koudelka C. W., Simpson E. L. (2013). Mental health comorbidity in patients with atopic dermatitis. *Journal of Allergy and Clinical Immunology*.

[28] Schmitt J., Romanos M., Schmitt N. M., Meurer M., Kirch W. (2009). Atopic eczema and attention-deficit/hyperactivity disorder in a population-based sample of children and adolescents. *The Journal of the American Medical Association*.

[29] Park G., Jung Y. S., Park M. K., Yang C. H., Kim Y. U. (2018). Melatonin inhibits attention-deficit/hyperactivity disorder caused by atopic dermatitis-induced psychological stress in an NC/Nga atopic-like mouse model. *Scientific Reports*.

[30] Jin W., Xu X., Chen X. (2019). Protective effect of pig brain polypeptides against corticosterone-induced oxidative stress, inflammatory response, and apoptosis in PC12 Cells. *Biomedicine & Pharmacotherapy*.

[31] Valvassori S. S., Resende W. R., Dal-Pont G. (2017). Lithium ameliorates sleep deprivation-induced mania-like behavior, hypothalamic-pituitary-adrenal (HPA) axis alterations, oxidative stress and elevations of cytokine concentrations in the brain and serum of mice. *Bipolar Disorders*.

[32] Kim Y., McGee S., Czeczor J. K. (2016). Nucleus accumbens deep-brain stimulation efficacy in ACTH-pretreated rats: alterations in mitochondrial function relate to antidepressant-like effects. *Translational Psychiatry*.

[33] Wang Y. L., Han Q. Q., Gong W. Q. (2018). Microglial activation mediates chronic mild stress-induced depressive-and anxiety-like behavior in adult rats. *Journal of Neuroinflammation*.

[34] Jin J. H., Lee D.-U., Kim Y. S., Kim H. P. (2011). Anti-allergic activity of sesquiterpenes from the rhizomes of cyperus rotundus. *Archives of Pharmacal Research*.

[35] Schinella G. R., Tournier H. A., Prieto J. M., de Buschiazzo P. M., Ríos J. L. (2002). Antioxidant activity of anti-inflammatory plant extracts. *Life Sciences*.

[36] Rocha F. G., Brandenburg M. d. M., Pawloski P. L. (2020). Preclinical study of the topical anti-inflammatory activity of cyperus rotundus L. extract (cyperaceae) in models of skin inflammation. *Journal of Ethnopharmacology*.

[37] Bae M. J., See H. J., Choi G., Kang C. Y., Shon D. H., Shin H. S. (2016). Regulatory T cell induced by Poria cocos bark exert therapeutic effects in murine models of atopic dermatitis and food allergy. *Mediators of Inflammation*.

[38] Feng D. D., Tang T., Lin X. P. (2016). Nine traditional Chinese herbal formulas for the treatment of depression: an ethnopharmacology, phytochemistry, and pharmacology review. *Neuropsychiatric Disease and Treatment*.

[39] Choi H.-J., Lee J.-H., Jung Y.-S. (2014). (+)-nootkatone inhibits tumor necrosis factor *α*-interferon *γ*-induced production of chemokines in HaCaT Cells. *Biochemical and Biophysical Research Communications*.

